# (*E*)-*N*-(3-(1-(2-(4-(2,2,2-Trifluoroacetamido)benzoyl)hydrazono)ethyl)phenyl)nicotinamide: A Novel Pyridine Derivative for Inhibiting Vascular Endothelial Growth Factor Receptor-2: Synthesis, Computational, and Anticancer Studies

**DOI:** 10.3390/molecules27227719

**Published:** 2022-11-09

**Authors:** Reda G. Yousef, Hazem Elkady, Eslam B. Elkaeed, Ibraheem M. M. Gobaara, Hanan A. Al-ghulikah, Dalal Z. Husein, Ibrahim M. Ibrahim, Ahmed M. Metwaly, Ibrahim H. Eissa

**Affiliations:** 1 Pharmaceutical Medicinal Chemistry & Drug Design Department, Faculty of Pharmacy (Boys), Al-Azhar University, Cairo 11884, Egypt; 2 Department of Pharmaceutical Sciences, College of Pharmacy, AlMaarefa University, Riyadh 13713, Saudi Arabia; 3 Zoology Department, Faculty of Science (Boys), Al-Azhar University, Cairo 11884, Egypt; 4 Department of Chemistry, College of Science, Princess Nourah bint Abdulrahman University, Riyadh 11671, Saudi Arabia; 5 Chemistry Department, Faculty of Science, New Valley University, El-Kharja 72511, Egypt; 6 Biophysics Department, Faculty of Science, Cairo University, Cairo 12613, Egypt; 7 Pharmacognosy and Medicinal Plants Department, Faculty of Pharmacy (Boys), Al-Azhar University, Cairo 11884, Egypt; 8 Biopharmaceutical Products Research Department, Genetic Engineering and Biotechnology Research Institute, City of Scientific Research and Technological Applications (SRTA-City), Alexandria 21934, Egypt

**Keywords:** pyridine, VEGFR-2 inhibitors, molecular docking, molecular dynamics simulations, DFT, ADMET, in vitro antiproliferative

## Abstract

(*E*)-*N*-(3-(1-(2-(4-(2,2,2-Trifluoroacetamido)benzoyl)hydrazono)ethyl)phenyl)nicotinamide (compound **10**) was designed as an antiangiogenic VEGFR-2 inhibitor with the essential pharmacophoric structural properties to interact with the catalytic pocket of VEGFR-2. The designed derivative was synthesized, and its structure was confirmed through Ms, elemental, ^1^H, and ^13^C spectral data. The potentiality of the designed pyridine derivative to bind with and inhibit the vascular endothelial growth factor receptor-2 (VEGFR-2) enzyme was indicated by molecular docking assessments. In addition, six molecular dynamic (MD) experiments proved its correct binding with VEGFR-2 over 100 ns. Additionally, the molecular mechanics energies, combined with the generalized born and surface area (MM-GBSA) analysis, identified the precise binding with optimum energy. To explore the stability and reactivity of the designed pyridine derivative, density functional theory (DFT) calculations, including electrostatic potential maps and total electron density, were carried out. Additionally, the absorption, distribution, metabolism, excretion, and toxicity (ADMET) analysis demonstrated its general likeness and its safety. The designed compound was synthesized to evaluate its effects against VEGFR-2 protein, cancer, and normal cells. The in vitro results were concordant with the in silico results, because the new pyridine derivative (compound **10**) displayed VEGFR-2 inhibition with an IC_50_ value of 65 nM and displayed potent cytotoxic properties against hepatic (HepG2) and breast (MCF-7) cancer cell lines with IC_50_ values of 21.00 and 26.10 μM, respectively; additionally, it exhibited high selectivity indices against the normal cell lines (W-38) of 1.55 and 1.25, respectively. The obtained results present compound **10** as a new lead VEGFR-2 inhibitor for further biological investigation and chemical modifications.

## 1. Introduction

Cancer is a deadly, life-threatening condition that is second only to cardiovascular illnesses as a cause of death [1]. In recent decades, cancer incidence and mortality rates have significantly climbed across the globe [2]. Despite beginning a while long ago, the quest for effective and safer novel antitumor drugs remains an active research area due to the systemic toxicity brought on by conventional nonselective chemotherapies and the emergence of resistance to the currently available anticancer medications [3]. Therefore, it remains vital to continue researching the development of new anticancer drugs with enhanced tumor selectivity, efficacy, and safety.

Serious side effects from nonselective chemotherapeutic drugs are well-known. Meanwhile, the specific biochemical abnormalities that cancer cells exhibit set them apart from normal cells. Anticancer agents developed to treat such abnormalities are more likely to be powerful and selective [4].

Vascular endothelial growth factor receptor-2 (VEGFR-2), a transmembrane tyrosine kinase receptor, is one of the most efficient targets in the treatment of cancer [5]. Cell proliferation, motility, adhesion, and angiogenesis are important steps that are orchestrated by VEGFR-2 [6]. Inhibiting the VEGFR-2 signaling cascade decreases the proliferation of various cancer cell types. This is carried out by giving cancer cells new blood that contains nutrients and oxygen (angiogenesis) [7]. Additionally, VEGFR-2 overexpression significantly aided in the spread metastasis of solid tumors [8]. VEGFR-2 levels were discovered to be relatively high in cancers such as breast cancer, prostate cancer, colon cancer, cervical cancer, kidney clear cell cancer, brain glioma, bladder carcinoma, pancreatic cancer, oral cancer, and ovarian cancer [9].

As a result, anticancer medications that inhibit VEGFR-2, such as sorafenib, regorafenib, pazopanib, sunitinib, tivozanib, and Lenvatinib, are selective and effective for many cancer types [10].

Our lab have presented several anticancer candidates with VEGFR-2-inhibitory potential, from diverse classes and derivatives, including thieno [2,3-*d*]pyrimidine [11], benzoxazole [12], pyridine [13] quinazoline [14,15,16], thiourea-azetidine [17,18], and quinoxaline-2 (1*H*)-one [19,20,21]. 

In this article, our team has employed previous backgrounds in computational (in silico) chemistry, as well as drug design and chemical synthesis, to disclose a promising pyridine analog with a specific VEGFR-2-prohibitory activity. The pyridine derivative was first proposed according to the features of VEGFR-2 prohibitions. Then, its VEGFR-2-prohibitory potential was examined by molecular docking, MD simulations, MM/GBSA, and DFT [22,23,24,25]. Next, the drug-likeness profile was computed by in silico ADMET and toxicity studies. Finally, the lead compound was synthesized and evaluated in vitro for VEGFR-2 inhibition, anticancer activity, and safety.

### Rationale

**Figure 1:**
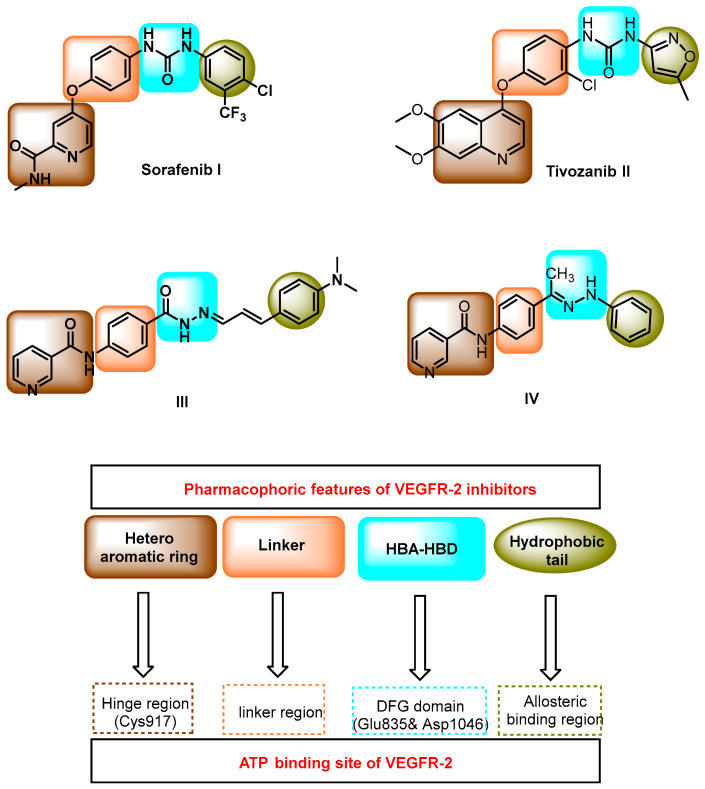
Some VEGFR-2 inhibitors and their essential pharmacophoric parts.

Sorafenib **I** [26] and tivozanib **II** [27] (Figure 1) are well-known VEGFR-2 inhibitors. Compounds **III** [28] and **IV** [29] were previously discovered by our team (Figure 1). These compounds are pyridine derivatives and exhibited promising antiproliferative VEGFR-2-inhibitory activities. In addition, these compounds exhibited an apoptotic effect.

Sorafenib **I**, tivozanib **II**, compound **III**, and compound **IV** share four essential pharmacophoric features required for good fitting with the VEGFR-2-binding sites, such as the ATP-binding site, the gatekeeper region, and the DFG motif region. Such pharmacophoric features comprise the following: (i) A hetero aromatic system to occupy the hinge region of the ATP-binding site. In this region, there is an essential amino acid (Cys917) that should be incorporated in the binding interaction. (ii) A linker group to occupy the gatekeeper region of the active site. (iii) A pharmacophore nucleus (a collection of HBD and HBA) to occupy the DFG motif region. The pharmacophore moiety should bind efficiently with Asp1044 and Glu883 to exert maximal activity. (iv) A terminal hydrophobic tail to occupy the allosteric binding pocket of the ATP-binding site [15,30,31].

**Figure 2:**
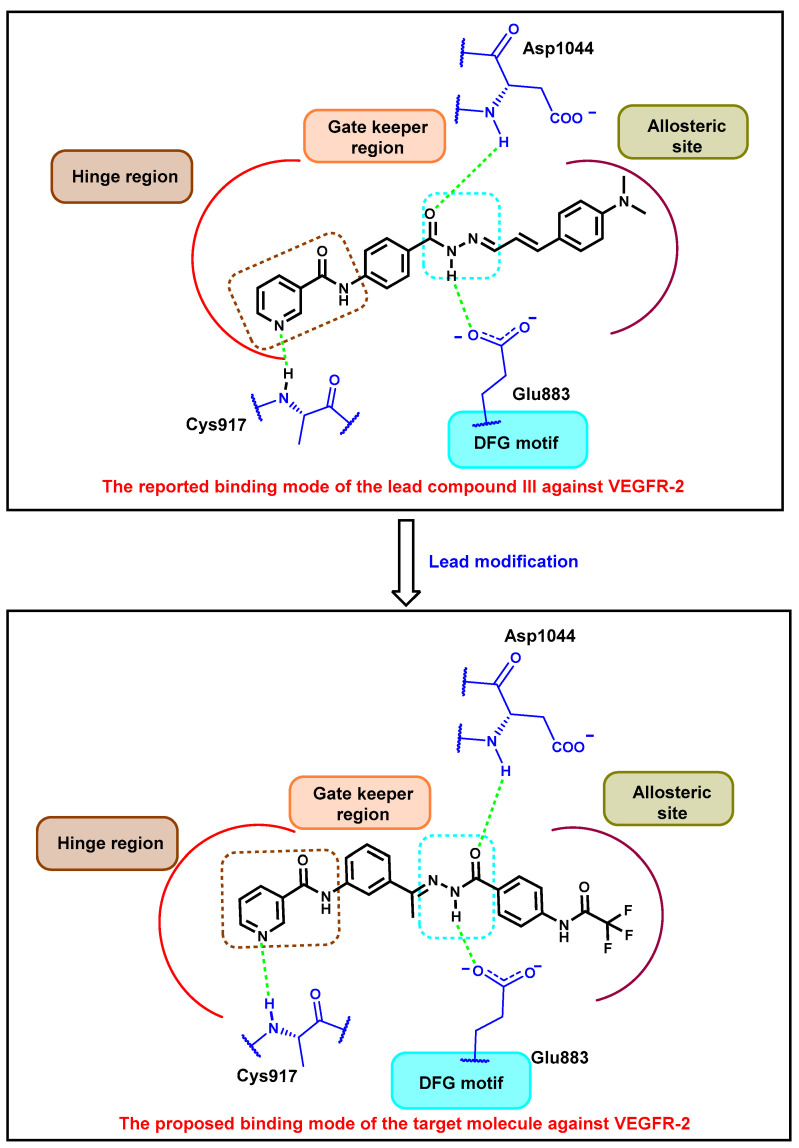
The design rationale of the targeted pyridine derivative.

In this work, compound **III** was used as a lead compound to discover a new VEGFR-2 inhibitor. Compound **III** was subjected to chemical modifications at two positions. The first position is the pharmacophore moiety, because we changed the orientation and bulkiness of the hydrazone moiety. We applied the same pharmacophore moiety of compound **V**. This modification may increase the hydrogen-bonding interaction at the DFG motif region. The second position is the terminal hydrophobic tail. We applied the extension strategy (addition of an extra function group). In this strategy, the *N,N*-dimethylamino group attached to the 4-postion of the terminal phenyl ring was substituted with the 2,2,2-trifluoroacetamide moiety. This modification may increase the chance of forming extra interactions in the allosteric binding site (Figure 2). Accordingly, the designed compound contains essential features to bind with and inhibit VEGFR-2.

## 2. Results and Discussions

### 2.1. In Silico Studies

#### 2.1.1. Molecular Docking

**Figure 3:**
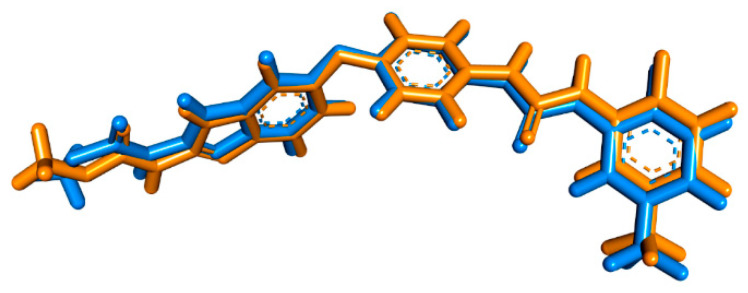
Validation step inside VEGFR-2 catalytic site; native ligand (**brown**) and the obtained pose (**blue**).

Molecular docking was performed to scrutinize the correctness of the carried-out design [32,33,34]. The validation of the docking algorithm was accomplished by re-docking the co-crystallized ligand in the active site of VEGFR-2 (PDB ID: 2OH4) [35,36]. The obtained RMSD between the docking pose and native crystallographic pose was 0.65 Å. This accepted value signposted the validity of the nominated docking algorithm (Figure 3).

**Figure 4:**
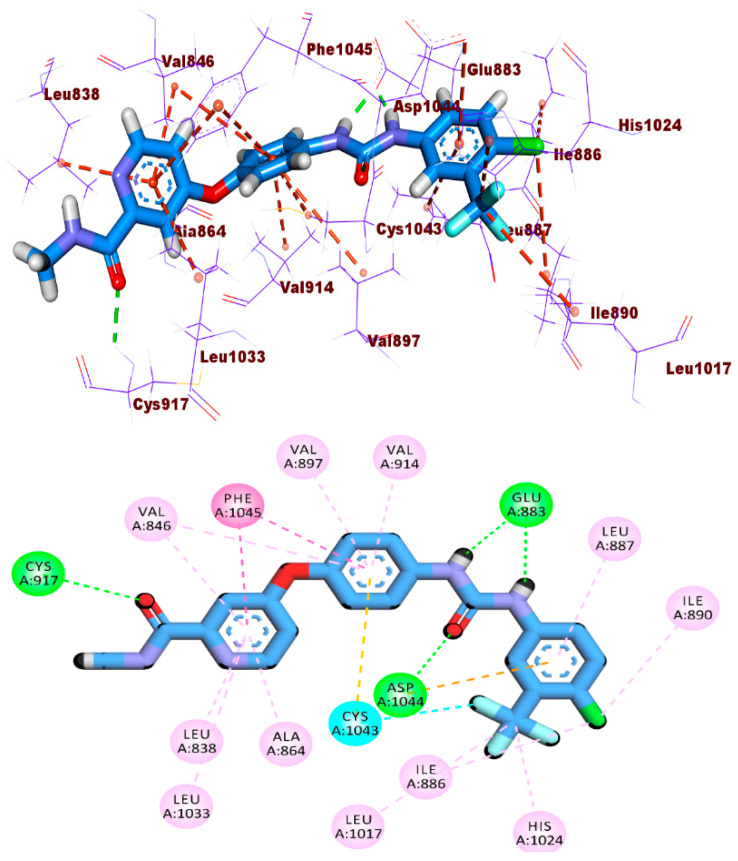
The 3D and 2D binding modes of sorafenib into VEGFR-2 active site.

Docking of sorafenib into the active site of VEGFR-2 was found to retrieve the reported binding mode [17,37,38] and reproduced a binding pose with a docking score of −20.19 kcal/mol. Sorafenib interacts using the NH of its pyridine scaffold with the hinge region residue Cys917. In addition, it achieved hydrophobic contacts with the hydrophobic pocket residues Leu887, Leu1017, Ile890, Ile886, Ile890, and Ala864. In the gatekeeper area, many hydrophobic interactions were observed with Val914, Val897, Phe1045, and Cys1043. The urea linkage finally served as a pharmacophore moiety and interacted with the key amino acid residues Asp1044 and Glu883 in the DFG motif (Figure 4).

**Figure 5:**
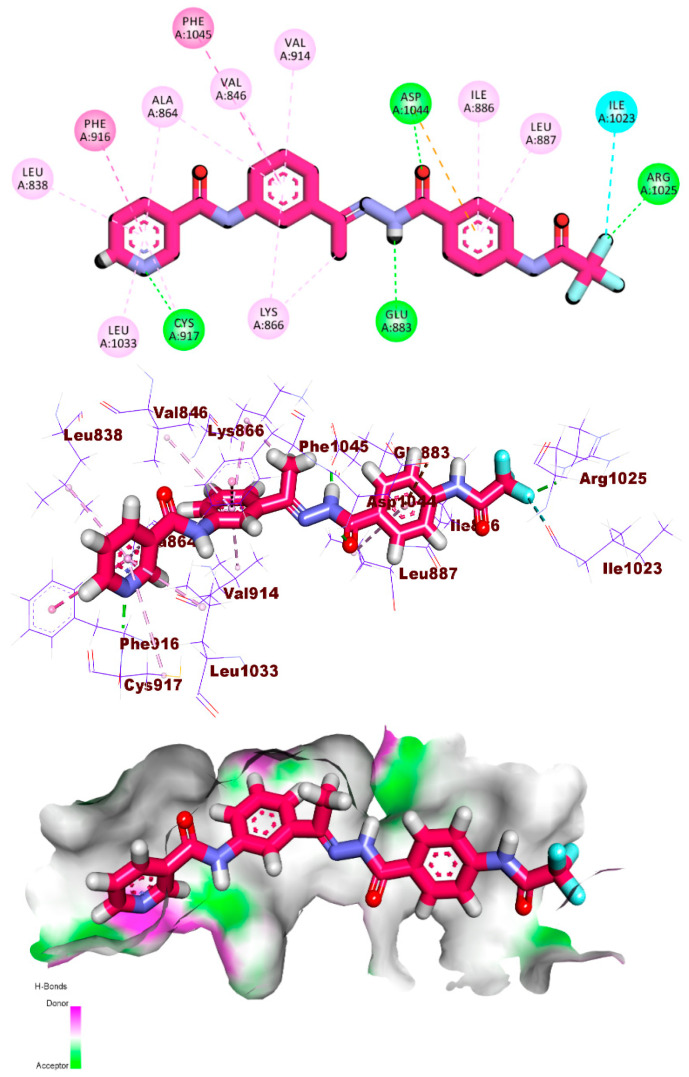
The 2D, 3D, and mapping surface binding patterns of the pyridine derivative compound **10** with the active site of VEGFR-2.

As planned in the rationale part, the analyzed compound **10** fitted well into the ATP-binding site of VEGFR-2 with an energy-binding score of -20.20 kcal/mol. The docked compounds showed a converged binding pattern similar to that of sorafenib. It is worth noting that the amide linkage of the pyridine arm functions as a pharmacophore moiety, forming two essential hydrogen bonds with Asp1044 (1.98) and Glu883 (1.77). Meanwhile, the pyridine moiety was also fitted into the hinge region to form a key hydrogen bond with Cys917 (2.11) and five hydrophobic bonds with Cys917, Leu838, Leu1033, Phe916, and Ala864. On the other hand, the designed pyridine derivative was stabilized in the linker region through its central phenylethylidene moiety via achieving five hydrophobic interactions with Lys866, Val914, Val846, and Phe1045. In addition, the 2,2,2-trifluoro-*N*-phenylacetamide arm was successfully buried in the allosteric binding region to form two hydrophobic bonds with Leu887 and Ile886 via the phenyl part. Additionally, in the same region, one hydrogen bond with Arg1025 and one halogen bond with Ile1023 were achieved through the 2,2,2-trifluoro arm (Figure 5). This network of interactions has the potential to improve binding affinity to VEGFR-2.

#### 2.1.2. MD Simulations

**Figure 6:**
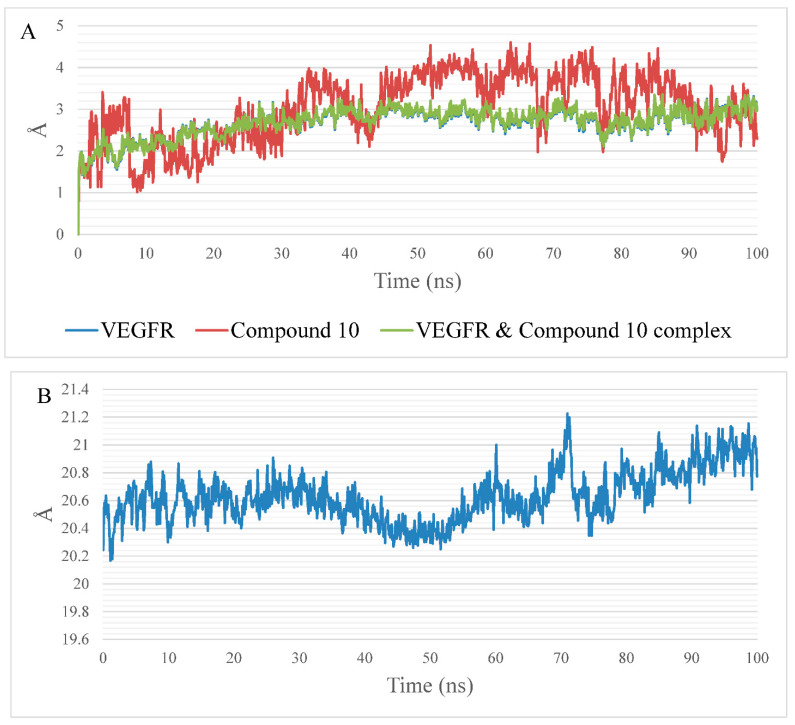

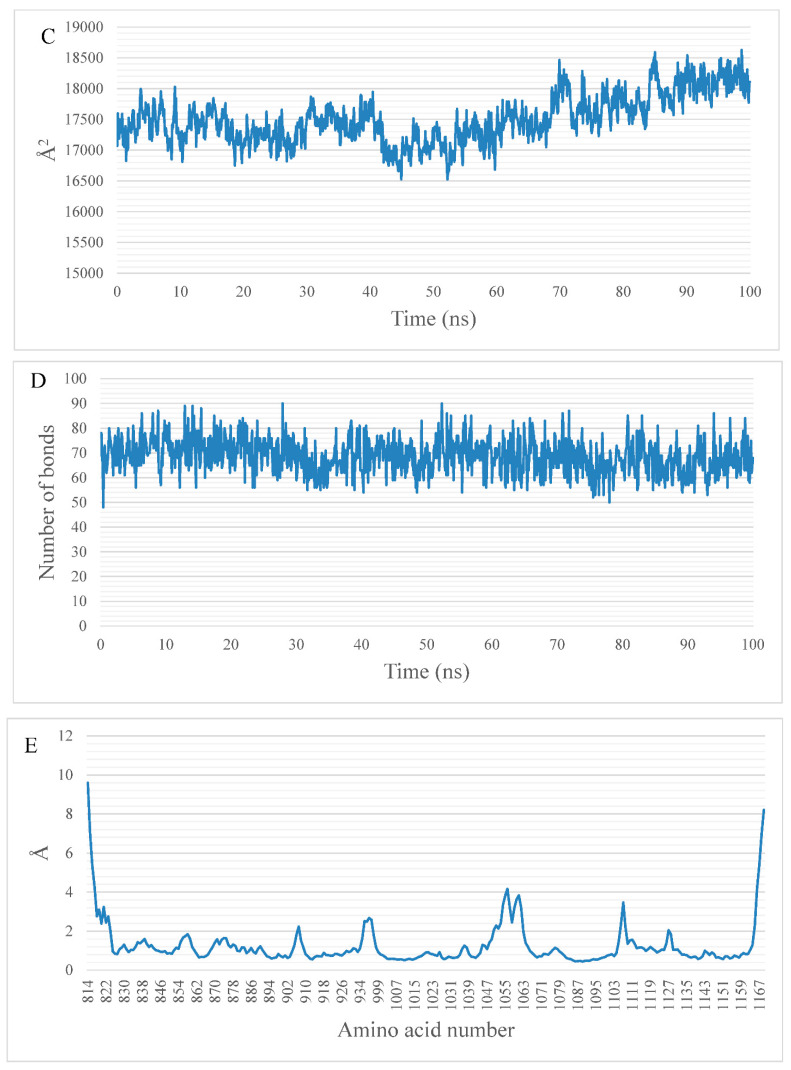

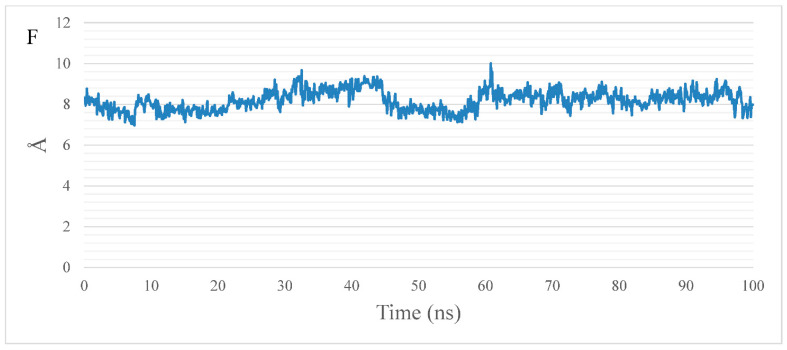
Measurements calculated from the MD trajectory: (**A**) RMSD—VEGFER-2 is the blue curve, compound **10** is the red curve, and complex is the green curve; (**B**) RoG; (**C**) SASA; (**D**) change in the number of H-bonds; (**E**) RMSF; and (**F**) center of the mass distance between compound **10** and VEGFR-2.

MD simulations were performed to validate the docking studies. The analyses performed on the production run show that the system equilibrated after approximately 30 ns. The RMSD plot (Figure 6A) showed a stable average after the first 30 ns at 2.6 Å for the protein (blue curve) and the complex (green curve). On the other hand, the RMSD of compound **10** (red curve) showed large fluctuations with an average of 3 Å. The RoG (Figure 6B), SASA (Figure 6C), and H-bonds (Figure 6D) showed a stable protein fluctuation with an average of 20.59 Å, 17,378 Å^2^, and 69 bonds. The fluctuation of the amino acids depicted in the RMSF plot (Figure 6E) showed low fluctuation (less than 2 Å) except for the free N-terminal, R1049:A1063, K1108:E1111, and the C-terminal, reaching 10 Å, 4 Å, 3.4 Å, and 8 Å, respectively. During the simulation, the ligand remained nearly in its place relative to the protein center of mass, with an average of 8.1 Å (Figure 6F).

#### 2.1.3. MM-GBSA Studies

##### Total Binding Energy and Its Decomposition Analysis

**Figure 7:**
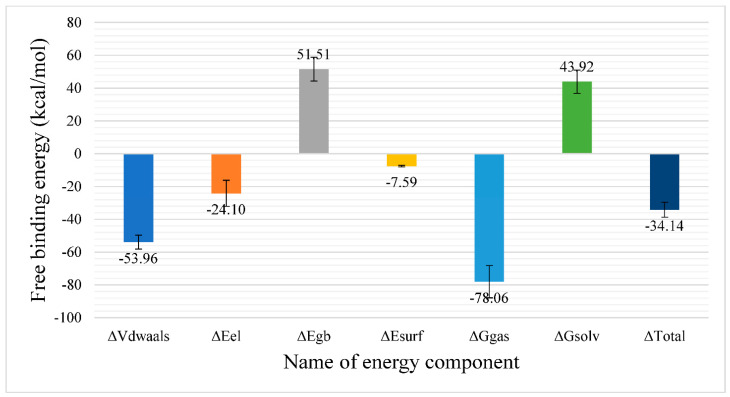
Energetic components of MM-GBSA and their average values. The bars represent the standard deviation of each energetic component.

**Figure 8:**
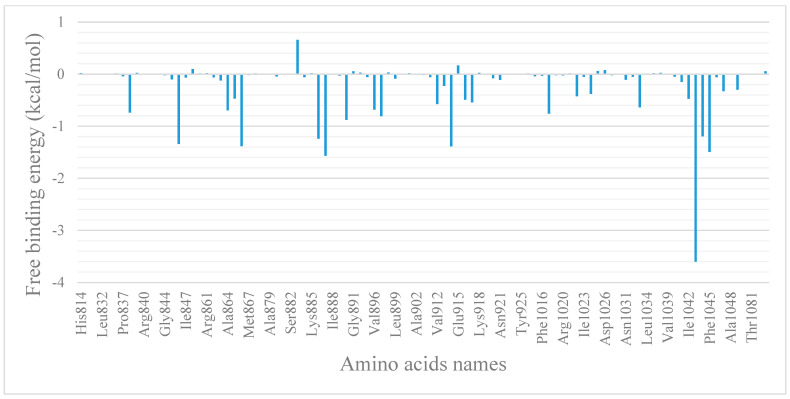
MM-GBSA binding free energy decomposition of the VEGFR-2–pyridinyl derivative complex. The figure shows the average contribution of each amino acid within 10 Å of the pyridinyl derivative compound.

Figure 7 shows the different components of the binding free energy analysis using MM-GBSA. Compound **10** showed a total binding with an average value of −34.14 Kcal/mol. The largest favorable contribution is the van der Waals interaction with an average value of −53.96 Kcal/mol followed by the electrostatic interaction, with an average value of −24.1 Kcal/mol. Moreover, we performed a decomposition analysis (Figure 8) to know which amino acids within 1 nm of the ligand have the highest contribution to the interaction. Val846 (−1.34 Kcal/Mol), Lys866 (−1.38 Kcal/Mol), Ile866 (−1.24 Kcal/Mol), Leu887 (−1.56 Kcal/Mol), Val914 (−1.38 Kcal/Mol), Cys1043 (−3.6 Kcal/Mol), Asp1044 (−1.19 Kcal/Mol), and Phe1045 (−1.49 Kcal/Mol) are the amino acids that have a contribution with a value better (less) than −1 Kcal/Mol.

##### Protein–Ligand Interaction Profiler (PLIP) Analysis

**Figure 9:**
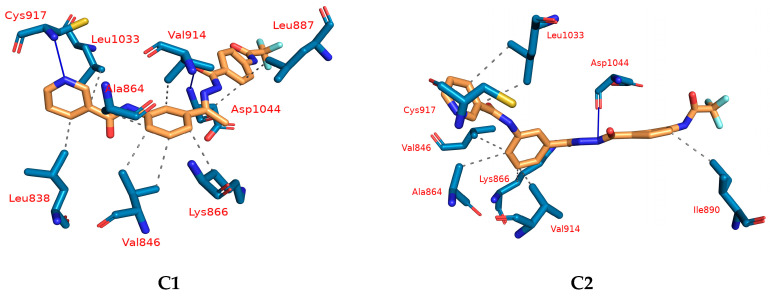

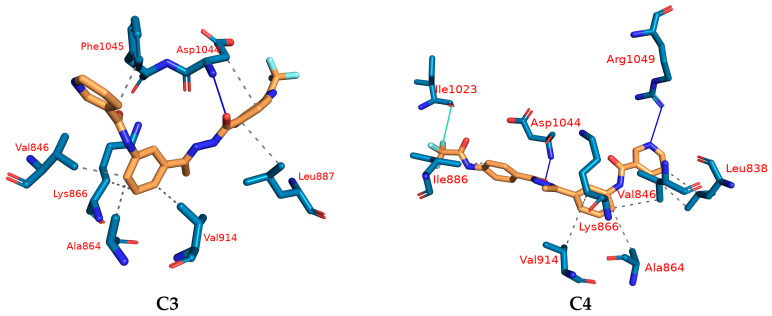
Types and number of different interactions produced from PLIP for each cluster representative. H-bond—blue solid line; hydrophobic interaction—dashed grey line; cyan solid line—halogen interaction; amino acids—blue sticks representation; compound **10**—orange sticks representation.

Next, the trajectory was clustered to obtain a representative frame for each cluster produced. As mentioned in the methods section, the number of clusters was selected automatically using the elbow method and this produced four clusters. For each cluster representative, PLIP webserver was utilized to determine the number and types of interactions between the ligand and the protein. Table 1 shows the number and types of interactions obtained from the PLIP webserver. The most common interaction is the hydrophobic interaction, with 30 interactions compared with 7 H-bonds in all cluster representatives. This is in line with the difference in the van der Waals and electrostatic energies values obtained from the MM-GBSA. Val846, Ala864, Lys866, and Val914 are the most common amino acids forming hydrophobic interactions in the four cluster representatives, while Asp1044 is the only common amino acid forming a H-bond. In the last cluster representative, Ile1023 forms a halogen bond with the fluorine atom. In addition to producing the interaction types and numbers from the PLIP webserver, it also generates a .pse file which visualizes the 3D conformation of the ligand and its interaction with the protein (Figure 9).

#### 2.1.4. DFT Calculations

##### Geometry Optimization and Mulliken Charge

**Figure 10:**
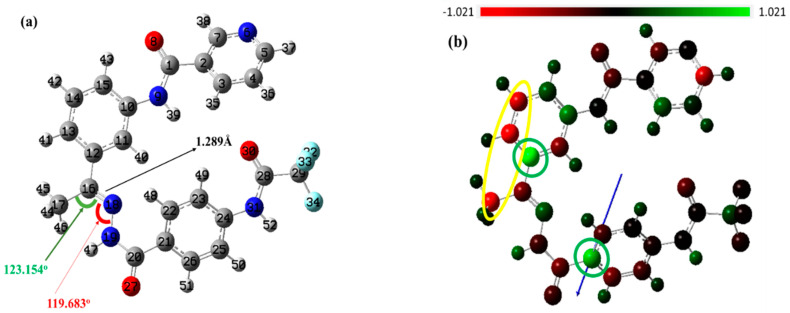
The fully optimized chemical structure (**a**), and the Mulliken charge (**b**) of the prepared compound at the B3LYB/6-311G++(d,p) level.

The molecule’s anticancer effect, which is controlled by its electronic chemical structure, has been explained by quantum computational DFT at the B3LYP/6-311++G (d, p) level. Figure 10 displays the ideal geometry after optimization and the color code for the atomic Mulliken charge distribution of the chosen heterocyclic molecule. In the 3D-gradient-optimized form shown in Figure 10a, the target molecule comprises a neutral singlet system made up of 52 atoms and 242 electrons. The condensation between compounds **9** and **4** produced compound **10,** with a C16-N18 bond length of 1.289 Å, and forms two angles of 123.154 Å (C17-C16-N18) and 119.683 Å (C16N18N19). 

The Mulliken charge analysis revealed the dipole moment, polarizability, negative or electron donor, and positive or electron acceptor charges of the selected anticancer drug. It also revealed how charges are distributed among the atoms in a molecule. The color scheme of Mulliken charge shown in Figure 10b denotes green for positive charge, red for the negative charge, and black for the neutral charge. As shown in Figure 10b, the most negative charges are distributed on the nicotinamide moiety, while most negative ones are located over the acetamide moiety. The dipole moment vector is shown and the calculated dipole moment for the optimized structure is 5.461 Debye.

##### Frontier Molecular Orbital (FMO) Analysis

**Figure 11:**
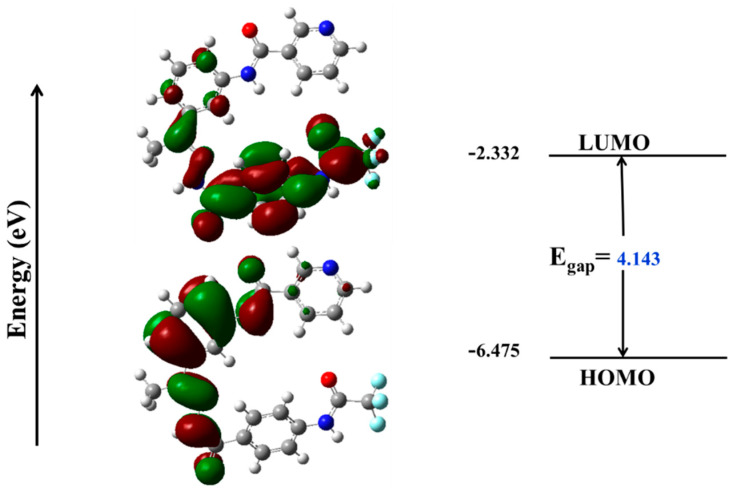
Molecular orbital surfaces and energy levels of the studied compound by using DFT calculations at the B3LYB/6-311G++(d,p) level.

The reactivity and stability of the compound’s structure can be associated in accordance with the FMO analysis of HOMO/LUMO energies (E_HOMO_ and E_LUMO_, respectively). The distributions of electrons over HOMO and LUMO are shown in Figure 11. The energy difference between HOMO and LUMO (E_gap_) affects the electronic properties of the anticancer inhibitor, as shown in Figure 11. The compound with a smaller E_gap_ value is considered to be more reactive when compared with a compound with a greater E_gap_ value. As can be seen in Figure 11, the DFT-conducted E_gap_ value is quite small, making the switch from the HOMO orbital to the LUMO orbital possible [39]. The estimated values for electron affinity (EA) and ionization potential (IP) are shown in Table 2. The chemical structure that was created has a fairly high electron affinity value, making it simpler to obtain electrons.

##### Chemical Reactivity Descriptors and Total Density of State (TDOS)

The FMO energy levels and E_gap_ are used to determine significant metrics, including chemical reactivity descriptors such as global hardness (η), maximal charge acceptance (N_max_), electronegativity (χ), chemical softness (δ), and electrophilicity (ω). Koopmans’ theory can be used to calculate these descriptors, as follows:

IP = −E_HOMO_

EA = −E_LUMO_

µ = (IP + EA)/2

η = (IP − EA)

χ = −η

ω = µ^2^/(2 η)

σ = 1/η

∆N_max_ = −(μ/η)

∆E = −ω

E_gap_= E_LUMO_ − E_HOMO_

where electronegativity (χ) assesses the Lewis acidity (the ability of a molecule to receive electrons), and global hardness (η) refers to a molecule’s ability to prevent the transfer of charge. Chemical softness (δ) quantifies the behavior of molecules toward electron transfer since the soft system has a smaller FMO E_gap_ and is better able to transmit its electrons to the acceptor system than the harder one. Based on the values in Table 2**,** it appears that the anticancer inhibitor could be reactive [40].

**Figure 12:**
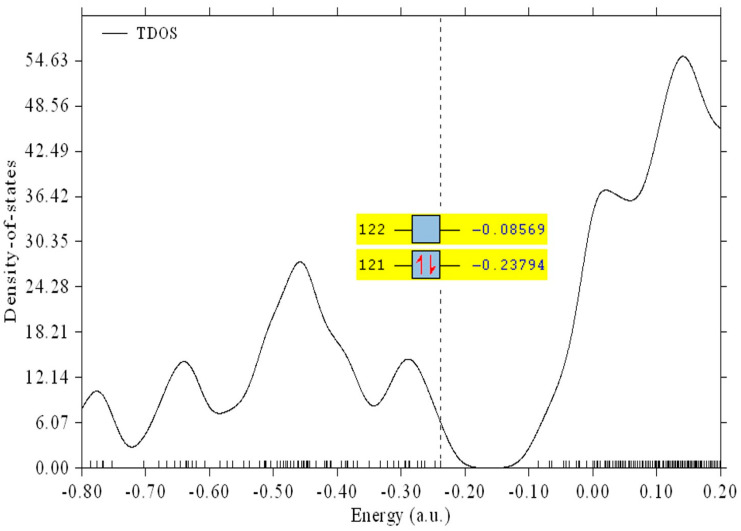
Molecular frontier orbital spectrum, their energies and total density of states (TDOS) were obtained at B3LYP/6-311++G(d,p).

The total density spectrum, displayed in Figure 12, was created using “the total density distribution function, TDOS,” because FMO analysis might not completely characterize electron density due to the potential of quasi-degenerate levels. The highest electronic density was recorded for orbitals higher than the LUMO orbital, as depicted in Figure 12.

##### Electrostatic Potential Maps (ESP)

**Figure 13:**
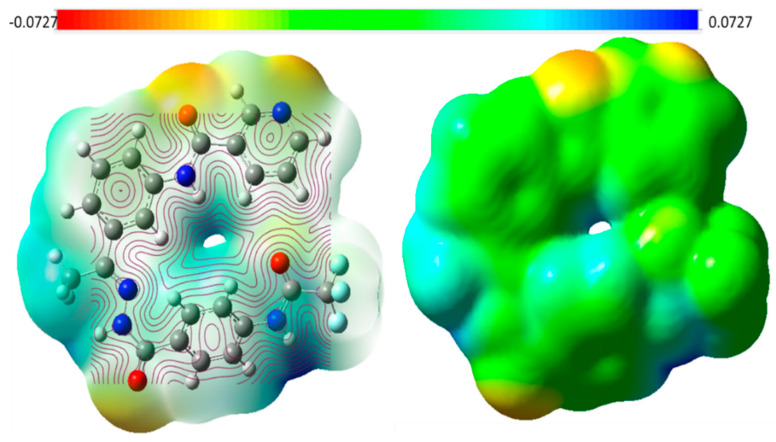
The electrostatic potential; ESP; maps of the selected compound at the 6-311G++(d,p) level.

The generated potential mapping of the synthesized heterocyclic molecule at the level DFT/B3LYP/6-311++G (d,p) is displayed in Figure 13. The ESP surface maps describe the intermolecular interaction and the behavior of the molecule toward the target. The ESP map demonstrates how oxygen atoms as expected represent active negative sites on the surface of the molecule. The most favorable reactive sites for nucleophilic attack are located over hydrogen atoms and are colored blue, while active sites for electrophilic attack are colored red on oxygen atoms. The fluoride ions are colored green, denoted as neutral active sites. The difference in electrical charge distribution may make the prepared anticancer medication a potential inhibitor.

#### 2.1.5. ADMET Profile Assessment

**Figure 14:**
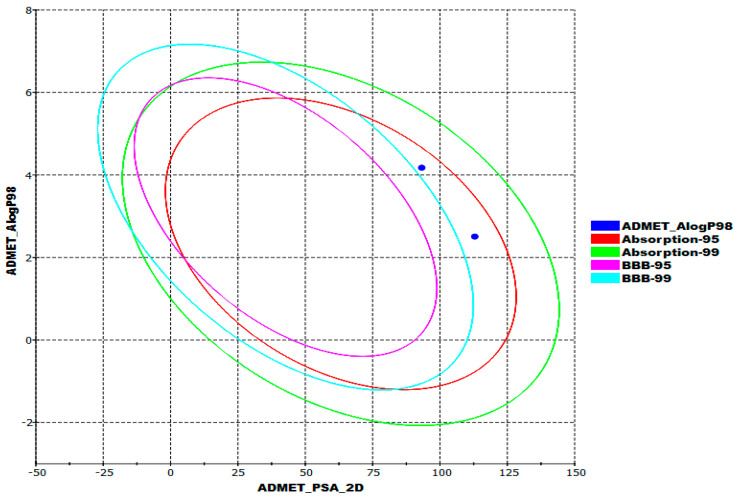
Computed ADMET parameters.

In addition to its biological activity, compound **10** has to be evaluated for its pharmacokinetic performance to be approved as a drug. So, any new compound should be evaluated for its ADMET properties at an early stage of drug development to reduce late drug withdrawals [41]. The ADMET model identifies the absorption, distribution, metabolism, excretion, and toxicity properties. There are various in vitro studies that can be performed to investigate ADMET properties, but in silico studies are more advantageous for a number of reasons, including cost, time, and effort limitations, in addition to strict regulations regarding animal testing [42]. The designed pyridinyl derivative was compared with sorafenib as a reference molecule, using Discovery Studio software to calculate ADMET parameters. Figure 14 demonstrates the examined ADMET profiles represented as ellipses: lipid–water partition coefficient (AlogP98, blue point); intestinal absorption (95% confidence limit (red ellipse) and 99% confidence limit (green ellipse); blood–brain barrier (BBB) (95% confidence limit (pink ellipse) and 99% confidence limit (turquoise ellipse). The two points lie outside the pink and turquoise ellipses and inside red and green ellipses explained that there were high degrees of similarities between the designed pyridine derivative and sorafenib in ADMET results (Figure 14). Both compounds had very low BBB transmembrane properties and good intestinal absorption levels, and neither compound was anticipated to inhibit the cytochrome P-450 (CYP2D6). The aqueous absorption and the ability to bind with plasma protein were computed to be low—less than 90% for the designed pyridine derivative and sorafenib. 

#### 2.1.6. In Silico Toxicity Assessment

An early toxicity assessment is of crucial importance in minimizing the failure of a drug in late development or during clinical trials [43]. Furthermore, the use of in silico approaches in toxicity prediction has become an essential part of drug development due to ethical codes, resource availability, and time wasted in conventional in vitro or in vivo studies [44]. In silico toxicity prediction is based on the structure–activity relationship (SAR)-predictive toxicity, where a computer compares the basic chemical structural properties of molecules with those of thousands of compounds that have either been reported to be safe or to be toxic (Table S1 in the Supplementary Materials) [45].

Based on the toxicity models built in the Discovery Studio software, nine parameters of acute and chronic toxicity were estimated computationally; the models employed were: FDA Rodent Carcinogenicity in female mice (FRC-FM); carcinogenic potential in mice as TD_50_ (TD_50_-M); developmental toxicity potential (DT-P); Ames Mutagenicity (A-M), which computationally determined whether the target compound has mutagenic potential or not; rat maximum tolerated dose and feed, (MTD-F); the oral LD_50_ value in rats (O-R- LD_50_); the chronic value of LOAEL in rats (C-LOAEL-R); the potential of irritancy against skin and eye. As illustrated in Table 3, our designed lead compound was computed to be safer than the reference drug (sorafenib).

### 2.2. Chemistry

**Scheme 1:**
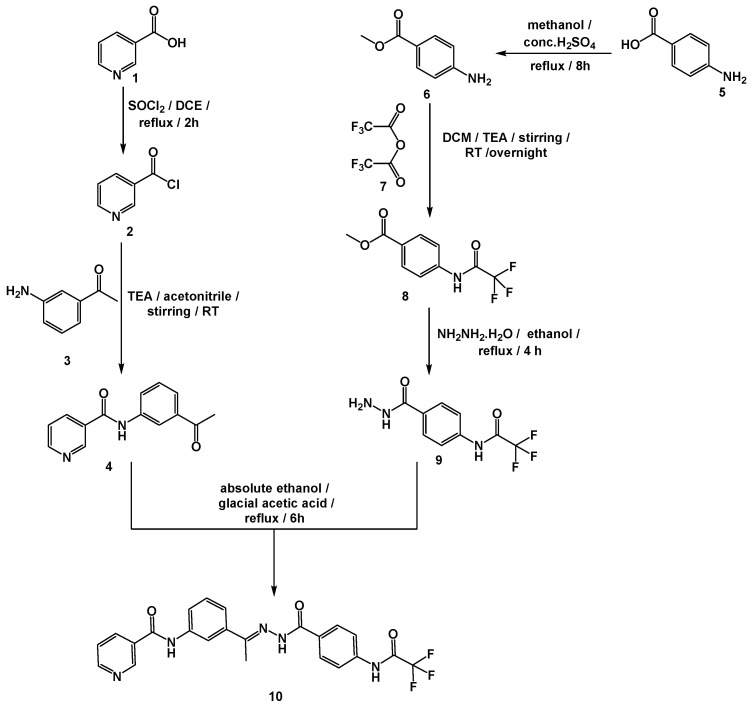
General procedure for the synthesis of the target—compound **10**.

To inspect the promising outputs of the in silico evaluations—which suggested that our designed pyridine derivative (compound **10**) had strong binding against VEGFR-2, and held general safety—compound **10** was prepared as outlined in the synthetic pathway (Scheme 1). The commercially available nicotinic acid **1** was successfully chlorinated using thionyl chloride to produce nicotinoyl chloride **2** in a good yield—exactly 80% [46]. The key nicotinamide derivative **4** was obtained later, via the reaction of nicotinoyl chloride **2** with 3-aminoacetophenone **3**. However, 4-aminobenzoic acid **5** was easily esterified by refluxing in a methanol/sulfuric acid mixture to generate the corresponding ester **6** [47]. Acylation of **6** with trifluoroacetic anhydride **7** in dichloromethane (DCM) at room temperature afforded methyl 4-(2,2,2-trifluoroacetamido)benzoate **8**. Compound **8** was then heated to reflux with hydrazine hydrate in absolute ethanol to obtain the key acid hydrazide derivative **9** [48]. Condensation of **9** with **4** afforded the final target—compound **10**—as presented in Scheme 1.

**Figure 15:**
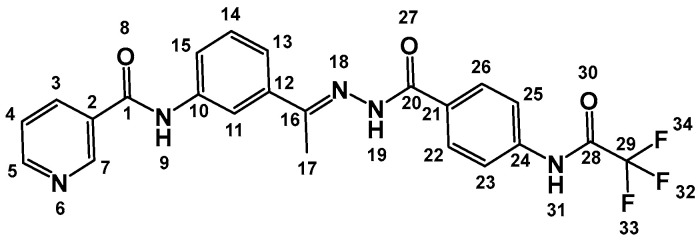
Chemical structure of compound **10**.

The IR spectrum (Figure S1 in the Supplementary Materials) of compound **10** was characterized by the appearance of carbonyl absorption bands at 1677 cm^−1^. The ^1^H NMR (Figures S2–S6 in the Supplementary Materials) revealed the presence of a characteristic 3H singlet signal at 2.35 ppm, corresponding to the CH_3_ group. Additionally, downfield singlet signals concerning the amidic protons have appeared at *δ* 10.62 and 10.30 ppm. The protons (3, 4, 5, and 7) of the pyridine ring (Figure 15) resonated at *δ* (8.67, 7.86, 8.90, and 9.32, respectively). Additionally, the meta-substituted benzene ring’s protons (11, 13, 14, and 15) appeared at (8.25, 7.43, 7.58, and 7.93 respectively). Furthermore, the di para substituted protons—protons (22, 26) and (23, 25)—resonated at *δ* 7.81 (d, *J* = 8 Hz) and *δ* 7.09 (d, *J* = 8 Hz), respectively. The validity of the proposed structure was also supported by ^13^C NMR spectra (Figures S7–S11 in the Supplementary Materials), which showed distinctive peak at 14.42 ppm, corresponding to the CH_3_ group.

### 2.3. In Vitro Biological Assessment

#### 2.3.1. VGFER-2 Inhibition

To examine the design, in addition to the obtained computational results—that solidly indicated the strong binding affinity of compound **10** to the VEGFR-2 enzyme—the inhibitory capability of compound **10** was estimated in vitro against the VEGFR-2-protein-contrasting sorafenib. Intriguingly, compound **10** firmly inhibited the VEGFR-2 enzyme with an IC_50_ value of 65.83 nM, which was close to sorafenib’s value (61.65 nM). The obtained in vitro outputs were harmonious with the acquired in silico results, and verified the strong potential of compound **10** to suppress VEGFR-2.

#### 2.3.2. Cytotoxicity

To adjudicate the efficiency of compound **10**’s VEGFR-2 prohibition against cancer, in vitro cytotoxicity assessment of compound **10** against HepG2 and MCF-7 malignant cell lines was performed, contrasting sorafenib as a reference drug. Table 4 illustrated the cytotoxic effects of compound **10** against HepG2 and MCF-7 cell lines, demonstrating IC_50_ values of 21.00 and 26.10 μM, respectively. The anticancer potentialities of compound **10** were almost tantamount to that of sorafenib (5.69 and 8.45 μM) against the same examined cell lines, respectively.

#### 2.3.3. Safety and Selectivity Index

To confirm the in silico safety results of compound **10** and identify its selectivity against cancer cell lines, the cytotoxic potential of compound **10** against the W-138 normal human cell line was investigated. 

Compound **10** expressed an excellent level of safety demonstrating a high IC_50_ value of 32.57 μM and very high selectivity indexes (SI) against the HepG2 and MCF-7 cell lines of 1.55 and 1.25, respectively. The obtained results indicated that compound **10** had near anticancer activity and much higher safety than sorafenib.

## 3. Experimental

### 3.1. In Silico Studies

#### 3.1.1. Docking Studies

Molecular docking was carried out using MOE2014 software. Detailed explanations are provided in the Supplementary Materials.

#### 3.1.2. M D Simulations

CHARMM-GUI webserver and GROMACS 2021 were utilized as an MD engine. Detailed explanations are provided in the Supplementary Materials.

#### 3.1.3. MM-GBSA

The Gmx_MMPBSA package was utilized. Detailed explanations are provided in the Supplementary Materials.

#### 3.1.4. DFT

Gaussian 09 and GaussSum3.0 programs were utilized. Detailed explanations are provided in the Supplementary Materials.

#### 3.1.5. ADMET Studies

ADMET profile was formulated using Discovery Studio 4.0. Detailed explanations are provided in the Supplementary Materials.

#### 3.1.6. Toxicity Studies

The toxicity profile was formulated using Discovery Studio 4.0. Detailed explanations are provided in the Supplementary Materials.

### 3.2. Chemistry

The solvents and fine chemicals used in the synthesis of the target molecule were purchased from Sigma-Aldrich, Darmstadt, Germany with purity of more than 99%. All chemicals and apparatus used in this section are illustrated in the Supplementary Materials.

#### General Procedure for the Synthesis of Compound **10**

2,2,2-Trifluoro-*N*-(4-(hydrazinecarbonyl)phenyl)acetamide **9** (0.001 mol, 0.25 g) and *N*-(3-acetylphenyl)nicotinamide **4** (0.001 mol, 0.24 g) were mixed and thoroughly dissolved in a round-bottomed flask containing absolute ethanol (25 mL). After that, the entire mixture was refluxed for 6 h while being catalyzed by drops of glacial acetic acid. The reaction was observed using TLC. The mixture was concentrated and cooled. Crystallization from methanol was used to filter and purify the collected product. 

((*E*)-*N*-(3-(1-(2-(4-(2,2,2-Trifluoroacetamido)benzoyl)hydrazono)ethyl)phenyl)nicotinamide):


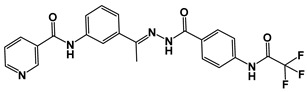


Off-white crystal (yield, 80%); m. p. = 246–248 °C; IR (KBr) *ν* cm^−1^: 3136 (NH), 3050 (CH aromatic), 2958, 2903 (CH aliphatic), 1677 (C=O); ^1^H NMR (400 MHz, DMSO-*d*_6_) δ 10.90 (s, 1H), 10.60 (s, 1H), 9.32 (d, *J* = 1.2 Hz, 1H), 8.90 (d, *J* = 4.8 Hz, 1H), 8.67 (d, *J* = 8.0 Hz, 1H), 8.25 (s, 1H), 7.27 (s, 1H), 7.93 (d, *J* = 8.2 Hz, 1H), 7.81–7.86 (m, 3H), 7.58 (dd, *J* = 8.0, 4.8 Hz, 1H), 7.43 (m, 1H, *J* = 8.2 Hz, 1H), 7.09 (d, *J* = 8.0, 2H), 6.71 (s, 1H), 2.39 (s, 3H); ^13^C NMR (101 MHz, DMSO-*d*_6_) δ 164.63, 152.65, 152.62, 149.20, 140.21, 135.99, 134.30, 130.98, 127.25, 123.99, 120.62, 120.30, 113.00, 14.42. Mass (*m*/*z*): 469 (M^+^, 15%), and 84.5 (100%, base peak); Anal. Calcd. for C_23_H_18_F_3_N_5_O_3_ (469.42): C, 58.85; H, 3.87; N, 14.92. Found: C, 58.97; H, 4.03; N, 15.18%. 

### 3.3. Biological Studies

#### 3.3.1. In Vitro VEGFR-2 Inhibition

In vitro VEGFR-2 inhibition was performed using a Human VEGFR-2 ELISA kit. Detailed explanations are provided in the Supplementary Materials.

#### 3.3.2. In Vitro Antiproliferative Activity

The MTT procedure was utilized to assess the in vitro antiproliferative activity. Detailed explanations are provided in the Supplementary Materials.

#### 3.3.3. Safety Assay

Normal cell lines W-138 were utilized in the safety assay. Detailed explanations are provided in the Supplementary Materials.

## 4. Conclusions

A pyridine-based derivative (compound **10**) was designed to be a VEGFR-2 inhibitor based on the essential structural properties of VEGFR-2 prohibitors. The anti-VEGFR-2 potentiality of the designed pyridine derivative was indicated by molecular docking and was confirmed by six MD (over 100 ns), three MM-GBSA, and three DFT experiments. Additionally, the ADMET analysis indicated the general likeness as well as safety. After synthesis and biological evaluation, the in vitro results were concordant with the in silico results; compound **10** displayed VEGFR-2 inhibition, with an IC_50_ value of 65 nM and cytotoxic properties against HepG2 and MCF-7 cell lines, with IC_50_ values of 21.00 and 26.10 μM, and with high selectivity indices of 1.55 and 1.25, respectively. According to the obtained results, compound **10** is a lead promising candidate for further in vivo, preclinical, and clinical studies, as well as for additional chemical modifications.

## Figures and Tables

**Table 1:** Number and types of interactions detected from PLIP webserver. Bold amino acids are the most common in all representatives.

Cluster Number	Number of Hydrophobic Interactions	Amino Acids in Receptor	Number of Hydrogen Bonds	Amino Acids in Receptor	Number of Halogen Bonds	Amino Acids in Receptor
**C1**	9	Leu838–**Val846** (2)–**Ala864**–**Lys866**–Leu887–**Val914**–Leu1033–Asp1044	2	Cys917–**Asp1044**	0	None
**C2**	7	**Val846**–**Ala864**–**Lys866**–Ile890–**Val914**–Leu1033 (2)	2	Cys917–**Asp1044**	0	None
**C3**	7	**Val846**–**Ala864**–**Lys866**–Leu887–**Val914**–Asp1044–Phe1045	1	**Asp1044**	0	None
**C4**	7	Leu838 (2)–**Val846**–**Ala864**–**Lys866**–Ile886–**Val914**	2	**Asp1044**–Arg1049	1	Ile1023

**Table 2:** The calculated global reactivity indices and energetic parameters for compound **10**.

IP	EA	μ (eV)	χ (eV)	η (eV)	σ (eV)	ω (eV)	Dm (Debye)	TE (eV)	∆Nmax	∆E (eV)
−6.458	−1.465	−3.961	3.961	2.497	0.400	19.592	7.500	−31539.8	1.587	−19.592

**Table 3:** In silico toxicity.

Comp.	FRC-FM	TD_50_-M Unit: mg/kg/day	DT-P	A-M	MTD-F *	O-R- LD_50_ *	C-LOAEL-R *	Skin Irritancy	Ocular Irritancy
Compound **10**	Non-Carcinogen	55.367	Non-Toxic	Non-Mutagen	0.095	1.810	0.100	Non-Irritant	Mild
Sorafenib	Carcinogen	19.236	Toxic	Non-Mutagen	0.089	0.823	0.005		

* Unit: g/kg body weight.

**Table 4:** In vitro VGFER-2 inhibition, cytotoxicity, and safety of the target compound **10**.

	HepG2 IC_50_ (µM)	SI (HepG2)	MCF-7 IC_50_ (µM)	SI (MCF-7)	W-38 IC_50_ (µM)	VEGFR IC_50_ (nM)
Compound **10**	21.00	1.55	26.10	1.25	32.57	65.83
Sorafenib	5.96	2.77	8.45	1.95	16.49	61.65

## Data Availability

Data are available with corresponding authors upon request.

## Supplementary Materials

The following supporting information can be downloaded at: https://www.mdpi.com/article/10.3390/molecules27227719/s1, Full detailed methods of (Molecular Docking, MD Simulations, MM-GBSA, DFT, ADMET, synthesis and in vitro studies). Also, the spectral data (Figure S1. IR spectrum of compound **10**, Figures S2–S6. ^1^H NMR spectra of compound **10** and Figures S7–S11. ^13^C NMR spectra of compound **10** ^13^C NMR) and the detailed toxicity reports (Table S1) of compound **10** and Sorafenib.

## Sample Availability

Compound **10** is available from the authors.
